# Qing Hua Chang Yin alleviates chronic colitis of mice by protecting intestinal barrier function and improving colonic microflora

**DOI:** 10.3389/fphar.2023.1176579

**Published:** 2023-07-27

**Authors:** Yuying Han, Liya Liu, Youqin Chen, Huifang Zheng, Mengying Yao, Liujing Cao, Thomas J. Sferra, Xiao Ke, Jun Peng, Aling Shen

**Affiliations:** ^1^ Clinical Research Institute, The Second Affiliated Hospital and Academy of Integrative Medicine, Fujian University of Traditional Chinese Medicine, Fuzhou, China; ^2^ Fujian Key Laboratory of Integrative Medicine in Geriatrics, Fujian University of Traditional Chinese Medicine, Fuzhou, China; ^3^ Department of Pediatrics, Rainbow Babies and Children’s Hospital, Case Western Reserve University School of Medicine, Cleveland, OH, United States; ^4^ Department of Gastroenterology, The Second People’s Hospital Affiliated to Fujian University of Traditional Chinese Medicine, Fuzhou, China; ^5^ Fujian Clinical Medical Research Centre of Chinese Medicine for Spleen and Stomach, Fuzhou, China

**Keywords:** Qing Hua Chang Yin, chronic colitis, pro-inflammatory cytokines, tight junction protein, intestinal microflora

## Abstract

**Background:** Qing Hua Chang Yin (QHCY) is a famous formula of traditional Chinese medicine (TCM) and has been proven to have protective effect on ulcerative colitis. However, its protective effect and potential therapeutic mechanisms in chronic colitis remain unclear. The purpose of this study is to explore the effects and underlying mechanisms of QHCY on dextran sulfate sodium (DSS)-induced chronic colitis mice model.

**Methods:** The chronic colitis model was established by administration of 2% DSS for three consecutive cycles of 7 days with two intervals of 14 days for recovery by drinking water. The experiment lasted 49 days. The DSS + QHCY group received QHCY administration by oral gavage at doses of 1.6 g/kg/d, DSS + Mesalazine group was administrated Mesalazine by oral gavage at doses of 0.2 g/kg/d. The control and DSS group were given equal volume of distilled water. The body weight, stool consistency and blood in stool were monitored every 2 days. The disease activity index (DAI) was calculated. The colon length was measured after the mice were sacrificed. The histomorphology of colonic tissues was checked by the HE and PAS staining. Immunohistochemistry was performed to detect the expressions of pro-inflammatory cytokines (TNF-α, IL-1β and IL-6), tight junction proteins (ZO-1, occludin) and Mucin2 (MUC2). 16S rRNA sequencing analysis was conducted to study the diversity and abundance of gut microbiota changes.

**Results:** QHCY treatment not only significantly attenuated DSS-induced the weight loss, DAI score increase, colon shortening and histological damage in mice, but also decreased the expression of pro-inflammatory cytokines in colonic tissues and increased the expression of ZO-1, occludin, and MUC2. Furthermore, QHCY enhanced the diversity of gut microbes and regulated the structure and composition of intestinal microflora in mice with chronic colitis.

**Conclusion:** QHCY has a therapeutic effect on a murine model of chronic colitis. It can effectively reduce the clinical and pathological manifestations of colitis and prevent alterations in the gut microbiota.

## 1 Introduction

Ulcerative colitis (UC) is a chronic inflammatory disease that affects the colon and rectum (Feuerstein et al., 2019). The main clinical symptoms of UC are weight loss, diarrhea, bloody mucopurulent stools (Ryan et al., 2020). During recent years, the incidence of UC has progressively increased (Ng et al., 2017). As the disease progresses, the risk of colitis-associated cancer increases significantly in UC patients (Shen et al., 2018). The commonly used drugs for the treatment of UC in clinic include: corticosteroids, 5-aminosalicylic acid, immunosuppressive agents (Yadav et al., 2016). However, patients receiving these therapeutics may develop adverse reactions and toxic effects to such a degree as to require discontinuation of medications (Yao H. et al., 2021). Additionally, these medications are costly and are not effective for every patient. Therefore, the development of efficacious, safe and low-cost effective drugs for the treatment of UC could benefit many patients.

The pathogenesis of UC has not been fully elucidated, but is acknowledged to be result of a combination of multiple factors (Actis et al., 2015). During the progression of UC, many pro-inflammatory cytokines play an important role in driving the inflammatory response, such as interleukin-6 (IL-6), interleukin-1β (IL-1β) and tumor necrosis factor-α (TNF-α) (Kim et al., 2020). Impaired mucosal barrier function is particularly important in the progression of UC (Chelakkot et al., 2018). The intestinal mucosal barrier consists of several components whose function are to prevent luminal contents, antigens, and pathogens from entering the blood stream (Wu et al., 2019). The tight junctions between intestinal epithelial cells are key components of the intestinal mucosal barrier (Yamada and Kanda, 2021). Occludin and ZO-1, as major epithelial tight junctions proteins, play an important role in maintaining the integrity of the intestinal mucosal barrier (Cui et al., 2020). MUC2, as an important component of the intestinal mucus barrier, can prevent contact between intestinal bacteria and the epithelial cells of the colon (Yao D. et al., 2021). Intestinal barrier function is closely related to intestinal flora, and dysbiosis of intestinal flora plays a crucial role in the pathogenesis of UC (Dalal and Chang, 2014). The imbalance of intestinal flora leads to the rapid growth and invasion of bacterial pathogens into intestinal epithelial cells, resulting in impaired intestinal mucosal barrier (Xu et al., 2020). Therefore, inhibiting the production of pro-inflammatory factors, stabilizing the intestinal mucosal barrier and maintaining intestinal flora may be potential therapeutic strategy for UC patients in clinical practice.

Traditional Chinese medicines (TCM) may have potential utility in treating UC (Ke et al., 2012). Qing Hua Chang Yin (QHCY) is a famous formula of TCM first prescribed by the academician Chun-bo Yang. It has a long history of use in China for the treatment of UC. QHCY mainly contains eleven traditional herbals, including *Herba et Gemma Agrimoniae*, *Radix Sanguisorbae*, *Radix Paeoniae Rubra*, *Coptis chinensis Franch*, *Magnolia officinalis*, *Elettaria cardamomum*, *Herba Eupaatorii Fortunei*, *Artemisia capillaris Thunb*, *Semen Dolichoris Album*, *Semen Coicis* and *Poria cocos*. Our previous studies showed that QHCY significantly attenuated clinical symptoms and pathological damage in a mouse model of acute intestinal inflammation through the reduction of pro-inflammatory cytokine production via the IL-6/STAT3 and TLR4/NF-κB signaling pathways (Ke et al., 2015; Ke et al., 2013b). However, whether QHCY alleviates chronic colitis in mice by protecting the function of intestinal mucosal barrier and improving the intestinal microflora remains to be further investigated.

In this study, we evaluated the protective effect of QHCY on DSS-induced the chronic colitis in mice. We aimed to provide a novel insight into the mechanisms of QHCY for the treatment of UC.

## 2 Materials and methods

### 2.1 Reagents and chemicals

The eleven traditional herbals in QHCY were provided by Beijing Tcmages Pharmaceutical Co., Ltd. DSS (M.W. 36000–50000) was purchased from MP Biochemicals (Solon, OH, United States). Mesalazine was purchased from Shanghai Aida Pharmaceutical Co., LTD (Shanghai, China). Hematoxylin was obtained from Solarbio Technology Co., Ltd. (Beijing, China). Antigen repair solution and phosphate buffer were purchased from Maixin Biotechnology (Fuzhou, Fujian, China). TNF-α (Cat. No. 41504) antibody was purchased from Signalway Biotechnology (St. Louis, MO, United States). IL-1β (Cat. No. 12242) antibody was obtained from Cell Signaling Technology (Beverly, MA, United States). Antibodies against IL-6 (Cat. No. ab208113), occludin (Cat. No. ab216327), ZO-1 (Cat. No. ab216880), and MUC2 (Cat. No. ab272692) were purchased from Abcam (Cambridge, MA, United States). Immunohistochemistry kits were purchased from Boster Biological Technology Co., Ltd. (Wuhan, Hubei, China).

### 2.2 Preparation of QHCY

QHCY formulation granules were prepared by Beijing Tcmages Pharmaceutical Co., Ltd. Briefly QHCY consists of eleven traditional herbals: 22 g dehydrated *Herba et Gemma Agrimoniae* (Cat. No.21016091), 10 g dehydrated *Radix Sanguisorbae* (Cat. No.21020571), 3.3 g dehydrated *Coptis chinensis Franch* (Cat. No.21013001), 11 g dehydrated *Radix Paeoniae Rubra* (Cat. No.21014551), 5.6 g dehydrated *Elettaria cardamomum* (Cat. No.21025151), 11 g dehydrated *Magnolia officinalis* (Cat. No.21017362), 11 g dehydrated *Artemisia capillaris Thunb* (Cat. No.21027521), 11 g dehydrated *Herba Eupaatorii Fortunei* (Cat. No.2101731), 22 g dehydrated *Semen Coicis* (Cat. No.21036561), 22 g dehydrated *Poria cocos* (Cat. No.21035691) and 11 g dehydrated *Semen Dolichoris Album* (Cat. No.21036561). As a first step in the preparation of QHCY, the 11 medicinal materials were extracted with distilled water. QHCY Chinese medicine formula granules were made by mixing the plant powders in an adequate dosage ratio and adding dextrin. QHCY was prepared with distilled water to a final concentration of 1.6 g/kg/day (200 μL solution per mouse). According to the “Pharmacological Experimental Methodology”, the dosage of QHCY for adult was calculated and then converted to the dose for mice by adjusting body surface area.

### 2.3 Animals and experimental protocols

**FIGURE 1:**
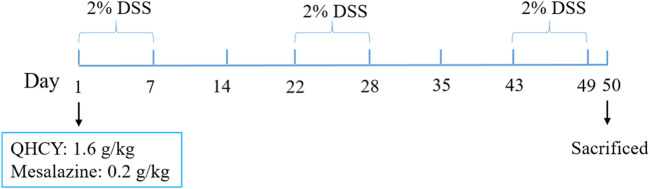
The schematic representation shows the experimental design and timeline of the mouse model.

The experimental animal procedures and protocols of this study were approved by the Animal Care and Use Committee of Fujian University of Traditional Chinese Medicine and the Animal Ethics Committee of Fujian University of Traditional Chinese Medicine (license number:2020097), and were strictly implemented in accordance with the Guidelines for the Care and Use of Laboratory Animals. C57BL/6 mice (Male, 25−30 g, 8–10 weeks) were purchased from SLAC Laboratory Animal Technology Co. Ltd. (Shanghai, China). All mice were kept under specific pathogen-free conditions with controlled temperature (22°C) and humidity, a 12 h light/dark cycle, and free access to food and water. Mice were randomly divided into four groups (n = 6): i) Control group; ii) DSS group; iii) DSS + QHCY group; iv) DSS + Mesalazine group. The chronic colitis model was established by administration of 2% DSS for three consecutive cycles (each is 7 days), with two intervals (each is 14 days) for recovery by drinking water. The experiment lasted 49 days (Wirtz et al., 2017) (Figure 1). The DSS + QHCY group received QHCY administration by oral gavage at doses of 1.6 g/kg/d, DSS + Mesalazine group was administrated Mesalazine by oral gavage at doses of 0.2 g/kg/d. The control and DSS groups were given equal volume of distilled water daily.

### 2.4 Evaluation of disease activity index (DAI)

The body weight of mice, stool consistency, and total blood in feces were monitored every 2 days. DAI of mice was obtained by combining the score of weight loss, fecal consistency and fecal blood, as shown in Table 1 (Wu et al., 2020). The colon was isolated after mice were sacrificed by cervical dislocation under anesthesia. Then colonic tissue and feces of mice were collected after the length of colon was measured.

**TABLE 1:** Disease activity index (DAI).

Index	Weight loss (%)	Stool	Crypt damage
0	None	Well-formed pellets	None
1	1–5	-	-
2	6–10	Pasty and semiformed	Positive bleeding
3	11–20	-	-
4	>20	Liquid	Gross bleeding

### 2.5 Histopathological analysis

The colonic tissue from each mouse was fixed in 4% paraformaldehyde and then embedded in paraffin after it was dehydrated in serially diluted solutions of ethanol and dimethyl benzene. The embedded tissues were cut into 4 µm sections. The sections were stained with hematoxylin and eosin (H&E) and periodic acid Schiff (PAS) after they were dewaxed and dehydrated. All specimens were observed at ×200 magnification using an optical microscope (Leica, Germany). The colonic tissue was evaluated for histological damage according to the score of inflammation, extent, crypt damage and percent involvement, as shown in Table 2.

**TABLE 2:** Histological grading of colitis in DSS-induced colitis mice.

Score	Inflammation	Extent	Crypt damage	Percent involvement (%)
0	None	None	None	None
1	Slight	Mucosa	Basal 1/3 damaged	1%–25%
2	Moderate	Mucosa and sub-mucosa	Basal 2/3 damaged	26%–50%
3	Severe	Transmural	Only surface epithelium intact	51%–75%
4			Entire crypt and epithelium lost	76%–100%

### 2.6 Immunohistochemical staining

Immunohistochemistry (IHC) was used to detect the expression of TNF-α, IL-1β, IL-6, occludin, ZO-1 and MUC2. Briefly, 4-μm-thick colon tissue slides were subjected to heat-induced antigen retrieval after dewaxing and hydration. After antigen retrieval, the sections were incubated with 3% H2O2 solution at room temperature for 15 min for blocking endogenous peroxidase activity. After the sections were blocked with 10% normal goat serum for 60 min, the tissues were incubated with rabbit polyclonal antibodies for TNF-α (1:200), IL-1β (1:50), IL-6 (1:50), occludin (1:100), ZO-1 (1:100), or MUC2 (1:500) overnight at 4°C, followed by HRP-conjugated secondary antibody (Maixin, Fuzhou, China) for 60 min at room temperature. A diaminobenzidine (DAB) kit (Maixin; Cat No. DAB-2031) was used for color development and the sections were counterstained with hematoxylin. The tissues on each slide were imaged under a light microscope (Leica, Germany) at ×400 magnification. And the percentage of positive cells from 6 fields was determined using ImageJ (open-source Java image processing program, https://imagej.nih.gov/ij/).

### 2.7 Microbial 16S rRNA gene sequencing and analysis

The cecal feces of mice were collected and the total bacterial DNA from fecal samples was extracted. Microbial 16S rRNA gene sequencing and analysis was performed by CapitalBio (Beijing, China). Primers were designed according to the conserved region, and sequencing connectors were added to the end of the primers for PCR amplification and production of sequencing libraries. The libraries were sequenced with Illumina NovaSeq.

### 2.8 Statistical analysis

Data obtained from experiments were analyzed using SPSS software 23.0 and expressed as the mean ± standard deviation (SD). One-way ANOVA was used for comparison between multiple groups (≥3) for data satisfying normal distribution and homogeneity of variance. When the normal distribution was not satisfied, nonparametric tests were used to analyze and compare data. Significant levels were fixed at *p* < 0.05.

## 3 Results

### 3.1 QHCY ameliorated clinical manifestations of DSS-induced chronic colitis

**FIGURE 2:**
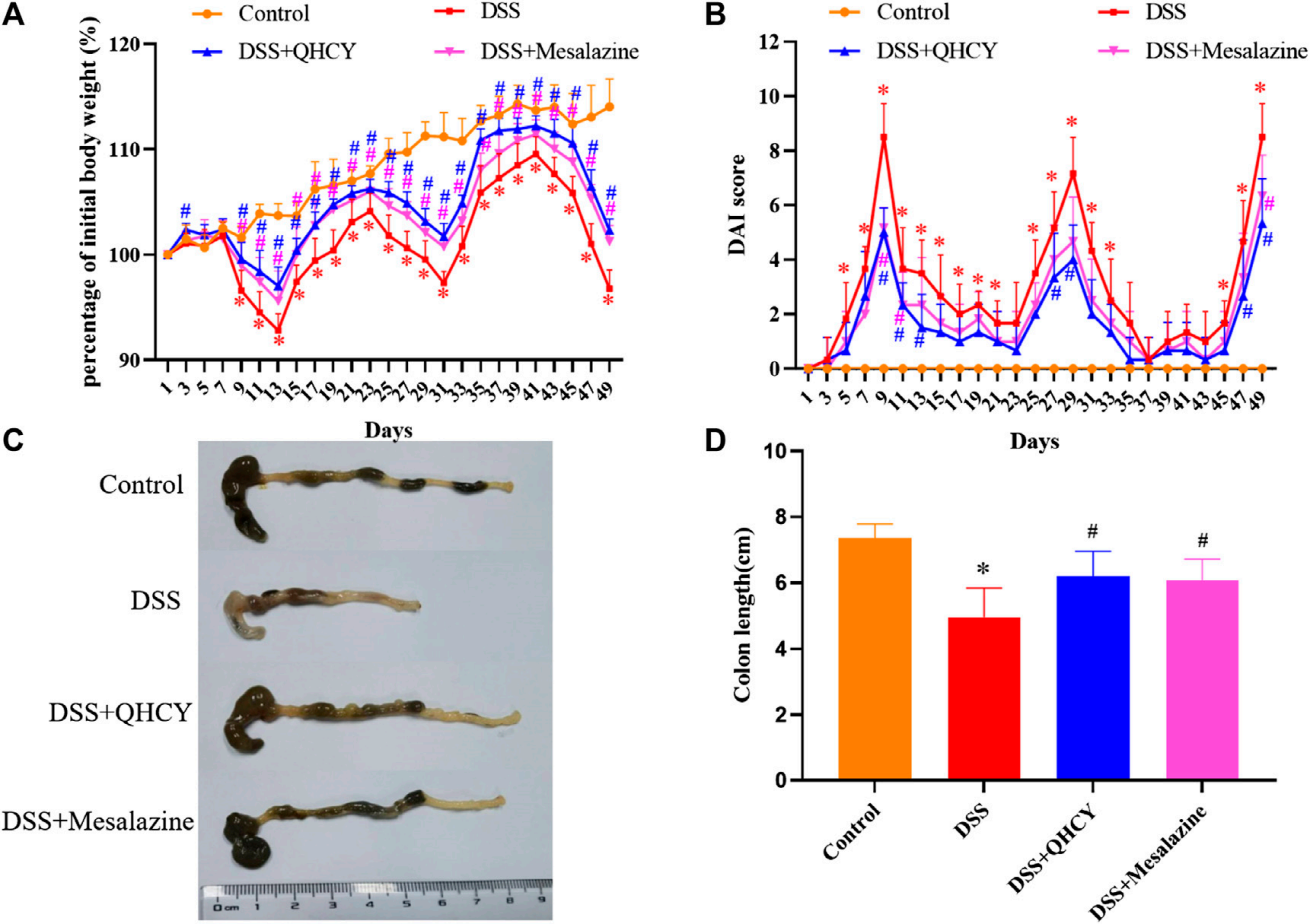
QHCY ameliorated clinical manifestations of DSS-induced chronic colitis. **(A)** Body weight was assessed every two days. **(B)** The DAI score was determined every two days. **(C)** Representative images of colon length. **(D)** Colonic length. ^*^ *p* < 0.05 vs the control group, ^#^ *p* < 0.05 vs the DSS group.

To determine the potential therapeutic efficacy of QHCY in chronic colitis of mice. We first evaluated body weight, DAI score, and colon length of mice. Compared with the control group, the DSS group showed a significantly weight loss (*p* < 0.05) (Figure 2A). The DAI scores increased markedly after DSS treatment (*p* < 0.05) (Figure 2B). As an indicator of the severity of colorectal inflammation, the colon length of mice was profoundly shortened in the DSS group compared with the control group (*p* < 0.05) (Figures 2C,D).However, QHCY treatment remarkably ameliorated DSS-induced the weight loss, DAI scores increase, and colon shorting in mice (*p* < 0.05). Consistent results were also observed in mice treated with Mesalazine.

### 3.2 QHCY ameliorated histological damage of colon tissue in the DSS-induced chronic colitis mice

**FIGURE 3:**
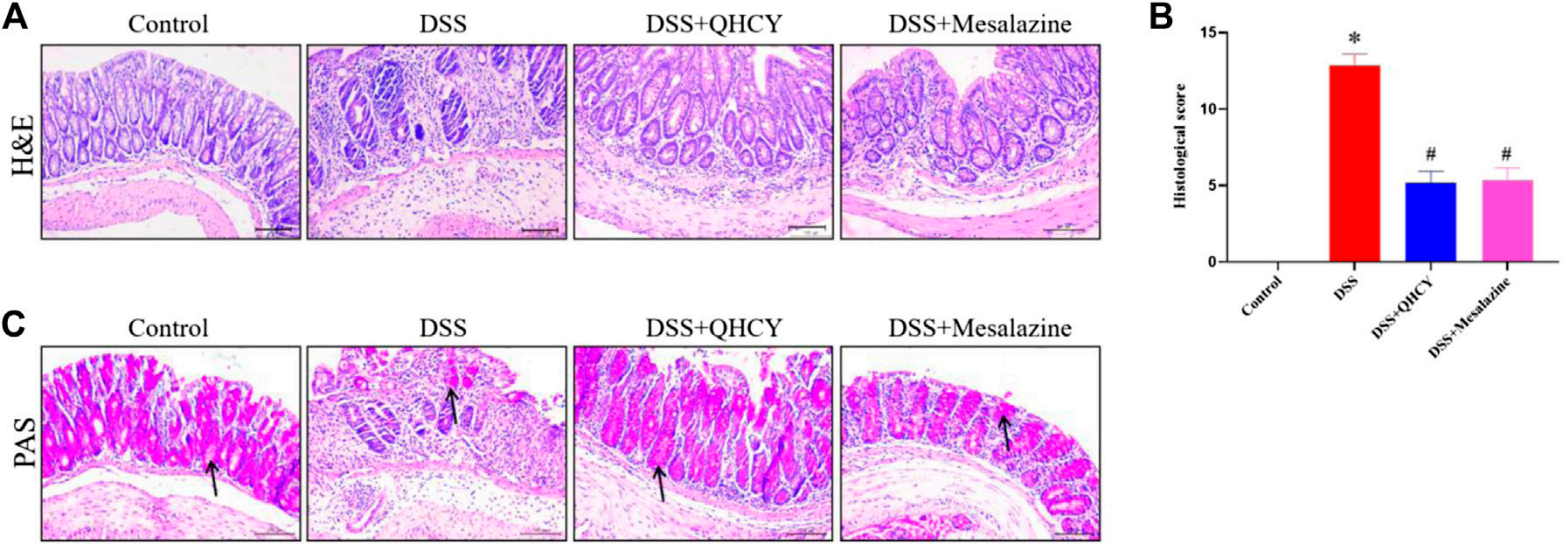
QHCY ameliorated histological damage of colon tissue in the DSS-induced chronic colitis mice. **(A)** H&E staining images of colonic tissues at ×200 magnification among four groups and **(B)** the histopathological score of colonic tissues. **(C)** PAS staining images of colonic tissues at ×200 magnification among four groups. *p < 0.05 vs the control group, ^#^ *p* < 0.05 vs the DSS group.

After H&E staining, the histological changes of the colonic mucosa of mice were imaged under a microscope. As shown in Figure 3A, the colon histology of mice in the control group was normal, with intact epithelium, clear glands, and no mucosal leukocyte infiltration. DSS induced mucosal ulceration, inflammatory cell infiltration, crypt deformation and epithelial cell proliferation in mice. However, QHCY treatment significantly ameliorated DSS-induced histological damage, which is similar as the results of Mesalazine treatment. Further histological scores analysis also showed that the histological scores in the DSS group were significantly higher than that in the control group (*p* < 0.05). QHCY and Mesalazine treatment obviously reduced DSS-induced the increase of degree of inflammation (*p* < 0.05) (Figure 3B). Compared with the control group, the DSS group exhibited varying degrees of atrophy in goblet cells, leading to reduced mucosal coverage. However, treatment with QHCY and Mesalazine, resulted in a reversal of intestinal mucosal epithelial cell and goblet cell atrophy to varying degrees, along with an increase in mucus secretion (Figure 3C).

### 3.3 QHCY reduced the expression of pro-inflammatory cytokines in DSS-induced chronic colitis

**FIGURE 4:**
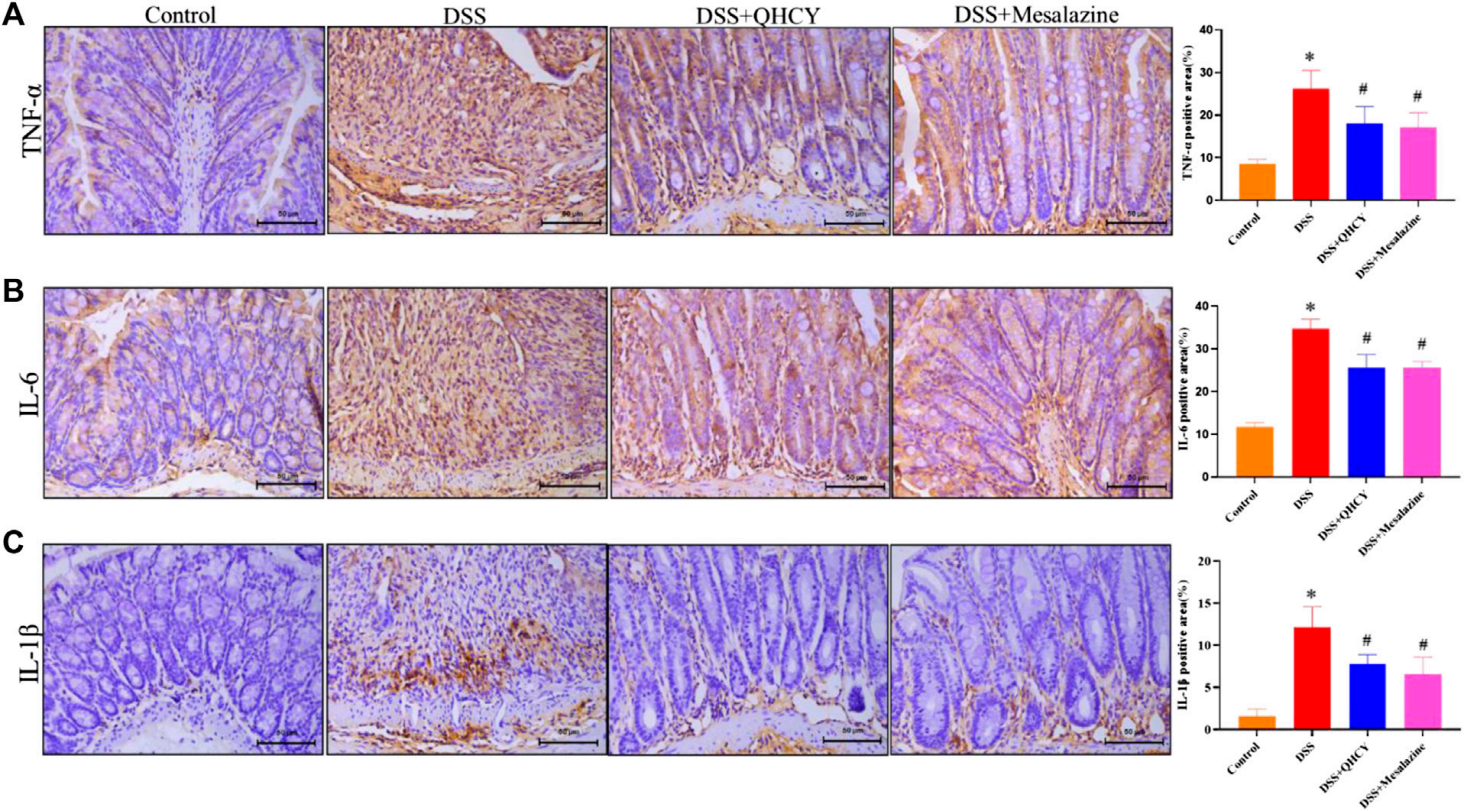
QHCY reduced the expression of pro-inflammatory cytokines in DSS-induced chronic colitis mice.Expression levels of proinflammatory cytokines, such as **(A)** TNF-α, **(B)** IL-6, **(C)** IL-1β in colon tissues. ^*^ *p* < 0.05 vs the control group, ^#^ *p* < 0.05 vs the DSS group.

To determine the anti-inflammatory potential of QHCY, IHC staining was employed to assess the expressions of pro-inflammatory cytokines in colonic tissues of mice. As shown in Figures 4A–C, the expression of TNF-α, IL-6 and IL-1β was significantly increased in the colon tissue of mice in the DSS group compared to the control group (*p* < 0.05). However, treatment with QHCY and Mesalazine notably suppressed DSS-induced the increase in the expression of these cytokines (*p* < 0.05).

### 3.4 QHCY restored intestinal mucosal barrier integrity in DSS-induced chronic colitis

**FIGURE 5:**
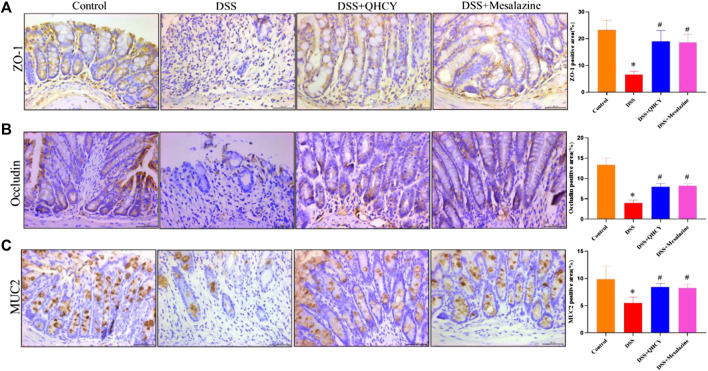
QHCY restored intestinal mucosal barrier integrity in DSS-induced chronic colitis mice. The expression of tight junction and mucin proteins: **(A)** ZO-1, **(B)** occludin and **(C)** MUC2 in the colon tissues was detected via immunocytochemistry. ^*^ *p* < 0.05 vs the control group, ^#^ *p* < 0.05 vs the DSS group.

Tight junctions (ZO-1 and occludin) and mucin (MUC2)-secreting proteins play an important role in maintaining the function of epithelial barrier. Therefore, we further analyze the expression levels of ZO-1, occludin and MUC-2 in colon tissues of mice by IHC staining. As expected, the expression of ZO-1, occludin and MUC2 were significantly decreased in the DSS group compared to the control group (*p* < 0.05). However, treatment with QHCY and Mesalazine profoundly inhibited DSS-induced the reduction in the expression of ZO-1, occludin, and MUC2 (*p* < 0.05) (Figures 5A–C).

### 3.5 QHCY changed the composition of gut microbes in DSS-induced chronic colitis

**FIGURE 6:**
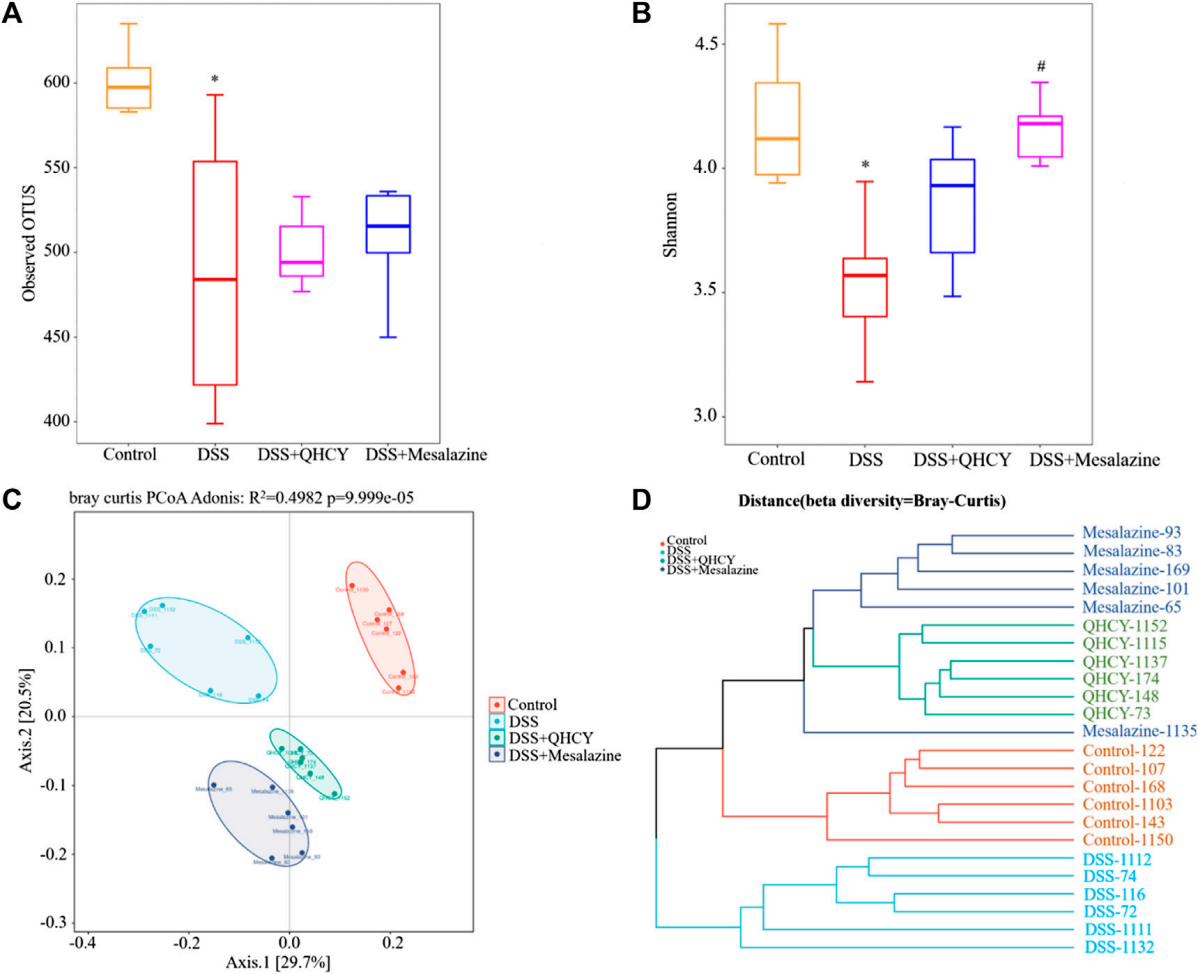
QHCY changed the composition of gut microbes in DSS-induced chronic colitis mice. **(A)** Observed OTUs **(B)** Shannon index **(C)** PCoA **(D)** Hierarchical clustering ^*^ *p* < 0.05 vs the control group, ^#^ *p* < 0.05 vs the DSS group.

Bacterial 16S rRNA was employed to assess the correlation between intestinal flora and the effect of QHCY administration during chronic colitis in mice. Compared with the control group, the number of operational taxonomic units (OTUs) in the DSS group was reduced (*p* < 0.05). This decrease was slightly improved after treatment with QHCY and Mesalazine, but the differences between the two groups were not statistically significant (*p* > 0.05) (Figure 6A). As shown in Figure 6B, the Shannon index was reduced in the DSS group compared with the control group (*p* < 0.05). QHCY slightly suppressed the change of Shannon index caused by DSS. But the difference was not statistically significant (*p* > 0.05). On the other hand, Mesalazine significantly inhibited the decrease of Shannon index induced by DSS (*p* < 0.05). Principal coordinate analysis (PCoA) and hierarchical clustering were applied to analyze the differences among microbial communities by beta-diversity. A significant separation was observed between the DSS group and the other three groups (Figure 6C). Hierarchical clustering analysis revealed that there were differences in the microbial community structure between the DSS and control group, and there were certain similarities in the composition of intestinal flora within the groups (Figure 6D).

**FIGURE 7:**
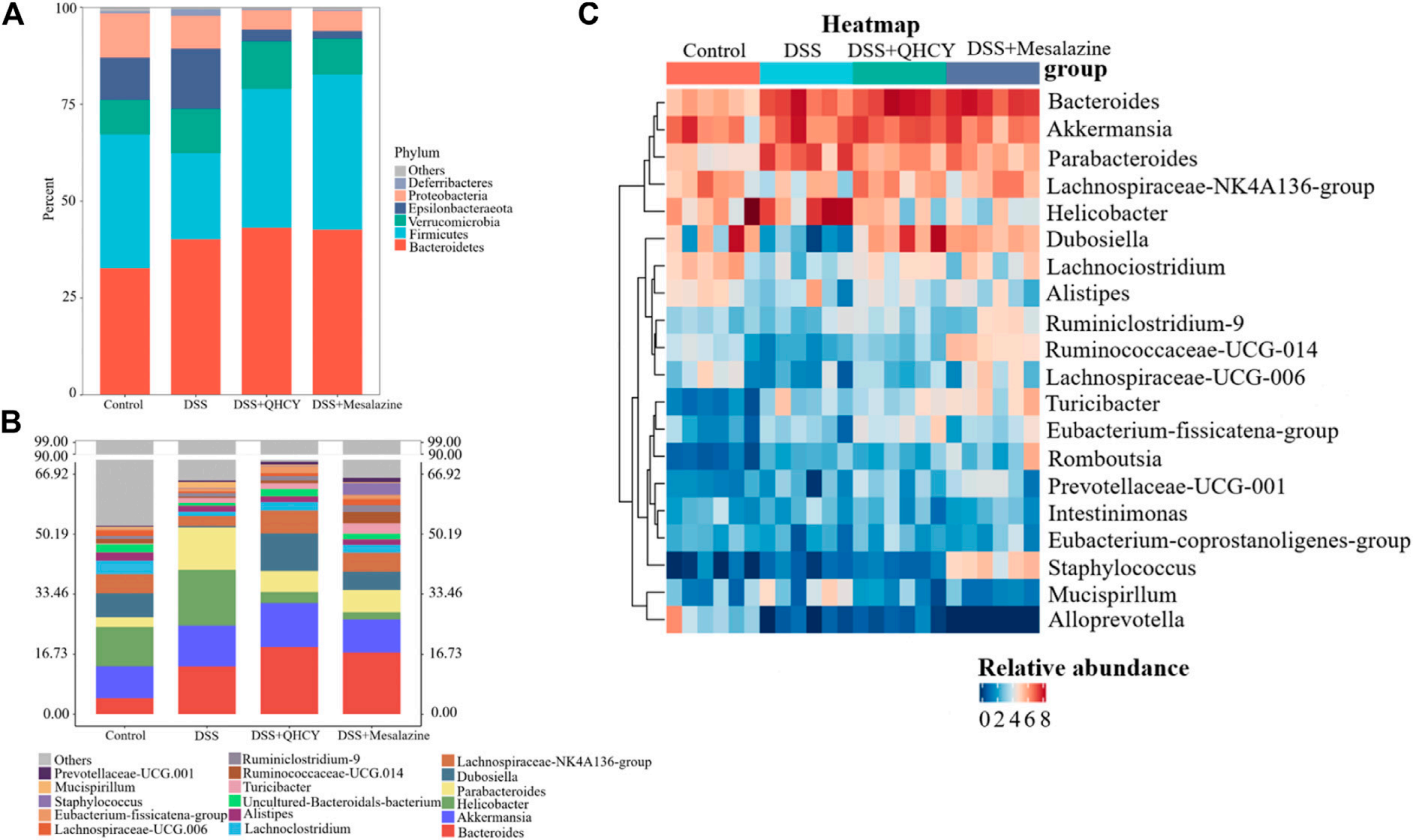
QHCY changed the composition of gut microbes in DSS-induced chronic colitis mice **(A)** Gut bacterial distribution at the phylum levels. **(B)** Gut bacterial distribution at the genus levels; **(C)** Heatmap represents the relative abundance of gut microbiota from mice at genus level.

Analysis of intestinal microbial composition showed that QHCY altered the abundance of intestinal microbial composition at phylum and genus levels. At the phylum level, *Bacteroidetes*, *Firmicutes*, *Epsilonbacteraeota*, *Verrucomicrobia* were the dominant phyla in the control group. DSS downregulated the relative abundance of *Firmicutes* and upregulated the levels of *Epsilonbacteraeota* compared with the control group, while QHCY and Mesalazine treatment reversed the effect of DSS on *Firmicutes* and *Epsilonbacteraeota* (Figure 7A). In addition, the ratio of *Firmicutes* to *Bacteroidetes* was reduced in the DSS group compared with the control group. However, the ratio of *Firmicutes* to *Bacteroidetes* for the QHCY and Mesalazine groups was increased compared to the DSS group (Figure 6E). The bacterial compositions of the four groups differed from each other at the genus level. Compared to the control group, increased *Helicobacter* and *Parabacteroides*, and decreased *Dubosiella* and *uncultured-Bacteroidales-bacterium* were observed in the DSS group. QHCY and Mesalazine exhibited regulatory effects on inhibiting *Helicobacter* and *Parabacteroides* and promoting *Dubosiella* and *uncultured-Bacteroidales-bacterium* (Figure 7B). In addition, the bacteria with significant differences among the experimental groups at the genus level were screened by heatmap. The abundance of *Dubosiella* was increased in the QHCY and Mesalazine group compared to the DSS group. Meanwhile, QHCY and Mesalazine decreased the abundances of *Helicobacter* in the mice of the DSS group (Figure 7C).

**FIGURE 8:**
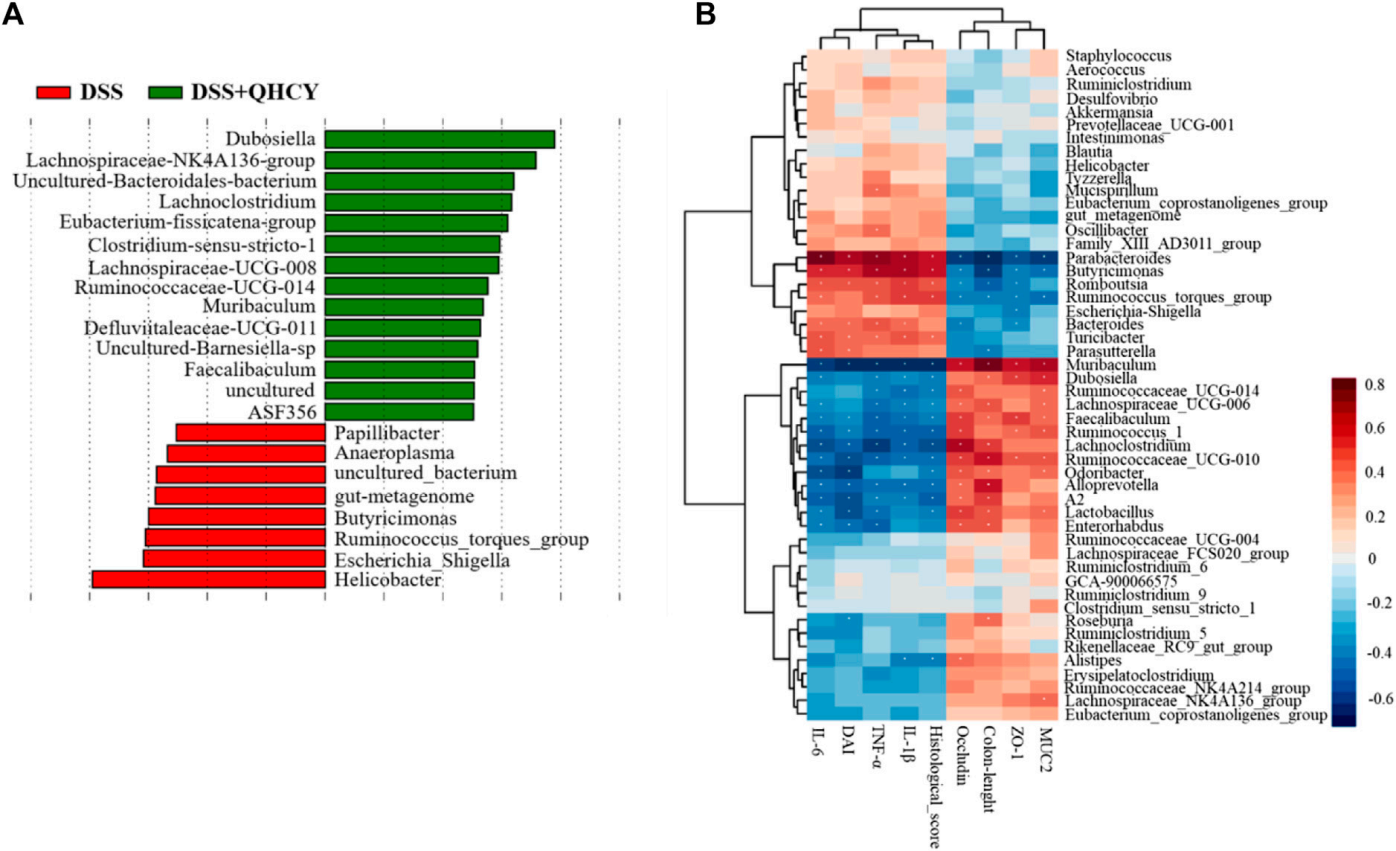
QHCY changed the composition of gut microbes in DSS-induced chronic colitis mice **(A)** The LEfSe analysis between DSS and DSS + QHCY group mice on the genus level. **(B)** Correlation analysis between the 50 most dominant genera in all samples and micro-environmental factors. Color grade shows the correlation degree. n = 6 for each treatment. *p < 0.05.

LEfSe analysis showed that *Helicobacter*, *Escherichia-Shigella*, *Ruminococcus-torques-group*, *Butyricimonas*, *gut-metagenome*, *uncultured-bacterium*, *Anaeroplasma* and *Papillibacter* were more abundant in the DSS group. The levels of *Dubosiella*, *Lachnospiraceae-NK4A136-group*, *Uncultured-Bacteroidales-bacterium*, *Lachnoclostridium*, *Eubacterium-fissicatena-group*, *Clostridium-sensu-stricto-1*, *Lachnospiraceae-UCG-008*, *Ruminococcaceae-UCG-014*, *Muribaculum*, *Defluviitaleaceae-UCG-011*, *Uncultured-Barnesiella-sp*, *Faecalibaculum*, *uncultured*, *ASF356* were more abundant in the DSS + QHCY group than in the DSS group (Figure 8A). Spearman correlation analysis was used to investigate the relationship between the 50 most abundant genera and DAI, colon length, histological score, pro-inflammatory cytokines (TNF-α, IL-1β and IL-6), tight junction proteins (ZO-1, occludin) and MUC2 (Figure 8B). *Parabacteroides*, *Butyricimonas*, *Romboutsia*, *Ruminococcus_torques_group*, *Bacteroides* and *Turicibacter* were positively related with pro-inflammatory cytokines (TNF-α, IL-1β and IL-6). *Muribaculum*, *Dubosiella*, *Lachnospiraceae_UCG-006*, *Faecalibaculum*, *Ruminococcus_1*, *Lachnoclostridium*, *Ruminococcaceae_UCG-010*, *Alloprevotella* and *A2* were negatively related with pro-inflammatory cytokines (TNF-α, IL-1β and IL-6). *Parabacteroides*, *Butyricimonas*, *Romboutsia* had a strong positive correlation with the DAI, histological score, but a negative correlation with colon length. *Muribaculum*, *Faecalibaculum*, *Lachnoclostridium*, *Ruminococcaceae_UCG-010*, *Odoribacter*, *Alloprevotella*, *A2* and *Lactobacillus* had a strong negative correlation with the DAI, histological score, but a positive correlation with colon length. *Parabacteroides*, *Butyricimonas* and *Ruminococcus_torques_group* were negatively related with ZO-1, occludin and MUC2. *Muribaculum*, *Faecalibaculum*, *Ruminococcus_1* and *Ruminococcaceae_UCG-010* were positively related with ZO-1, occludin and MUC2.

## 4 Discussion

QHCY is reported to exert a therapeutic effect in acute colitis (Ke et al., 2013a; Ke et al., 2013b). However, its protective effect in chronic colitis remains large unknow. In our study, we for the first time evaluated the protective effect of QHCY on chronic colitis of mice induced by DSS and the mechanisms by which it might act. Our results indicated that QHCY has a significant efficacy in the treatment of DSS-induced chronic colitis. QHCY attenuated the clinical symptoms of chronic colitis, such as preventing weight loss, reducing diarrhea and rectal bleeding. In addition, QHCY profoundly alleviated colonic shortening and pathologic damage of mice with chronic colitis. These findings for the first time confirmed that QHCY has a protective effect on DSS-induced chronic colitis of mice.

Inflammatory cytokines play an important role in the pathogenesis of colitis. TNF-α mediates intestinal mucosal injury and necrosis by amplifying the inflammatory cascade within the intestinal mucosa (Lin et al., 2019). IL-6 is a pleiotropic cytokine that is elevated in UC (Cao et al., 2021) and plays a critical role in the pathogenesis of UC by stimulating neutrophil infiltration (Gupta et al., 2018). IL-1β aggravates intestinal inflammation by damaging the intestinal TJ barrier and increasing intestinal permeability (Kaminsky et al., 2021). Our previous studies showed that QHCY significantly inhibited the LPS-induced secretion of pro-inflammatory TNF-α, IL-6 and IL-8 in the Caco-2 cells in a dose-dependent manner (Ke, Chen, et al., 2013; Ke et al., 2015). These results lead us to hypothesis that QHCY may alleviates chronic colitis by inhibiting inflammatory response in DSS-induced chronic colitis in mice. As expected, we found that DSS-induced elevation of TNF-α, IL-6, and IL-1β expressions in the mice were inhibited by QHCY treatment. These findings suggest that QHCY ameliorates chronic colitis by suppressing DSS-induced the expressions of pro-inflammatory mediators.

Both occludin and ZO-1 are key tight junction proteins involved in permeability, tissue differentiation, and homeostasis of the intestinal epithelium (Amasheh et al., 2009). Disruption of the tight junction barrier of the colonic epithelium is an important pathogenic factor promoting the development of chronic colitis (Wang et al., 2016).The mucus layer is also an essential component of the intestinal mucosal barrier, which effectively separates the intestinal epithelium from the intestinal lumen and protects the epithelial cells from stimulation by intestinal bacteria and food antigens (Yao D. et al., 2021; Yao H. et al., 2021). MUC2 is the most vital component of the mucus layer and its expression level is reduced in ulcerative colitis (Melhem et al., 2021). Our previous study showed that QHCY not only prevented the LPS-induced decrease of ZO-1, occludin and claudin-1 expression in the Caco-2 cells, but also prevented the decrease of these tight junction protein expressions in colonic tissues of acute colitis of mice (Ke et al., 2019; Fang et al., 2021). These results lead us to hypothesize that QHCY may alleviates chronic colitis by preventing disruption of the intestinal mucosal barrier integrity in DSS-induced chronic colitis in mice. As expected, we found that DSS-induced reduction of these tight junction protein expressions in the mice were prevented by QHCY treatment. These findings suggest that QHCY exert its therapeutic effect by regulating the expression of occludin, ZO-1, and MUC2.

Gut microbiota is comprised of trillions of bacteria involved in physiological functions related to nutrition, immune response, and the defense of the host (Nishida et al., 2018). The imbalance of intestinal flora play a critical role in the pathogenesis of chronic colitis (Goldsmith and Sartor, 2014). Thus, we investigated for the first time the effect of QHCY on the gut microbiota of mice with chronic colitis. Our results demonstrated that QHCY may enhance microbial community diversity. DSS reduced Shannon index compared with the control group. However, QHCY may have a tendency to inhibit the change of Shannon index. In addition, the date of OTUs displayed an increasing trend of microbial community diversity after QHCY treatment. Through PCoA and hierarchical cluster analysis, we found the changes in microbial community structure in the DSS group. The microbial community structure after QHCY treatment was different from that of the DSS group.

We further performed a more detailed taxonomic analysis of microbial community composition at phylum and genus level. At the phylum level, *Firmicutes* and *Bacteroidetes* dominate the gut microbial community. Previous work has shown that the proportion of *Firmicutes* is inversely correlated with gastrointestinal inflammation (Quévrain et al., 2016). It was reported that the ratio of *Firmicutes* to *Bacteroidetes* was reduced in mice with colitis (Cui et al., 2021), which was consistent with our research. Nevertheless, QHCY restored the dysfunctional gut flora to normal levels. *Epsilonbacteraeota* infected and proliferated within the intestinal tract of various animal hosts, resulting in various disease outcomes (Kelley et al., 2021). Thus, QHCY could alleviate chronic colitis by decreasing the relative abundance of gut barrier-harmful bacteria including *Epsilonbacteraeota*. At the genus level, QHCY treatment upregulated the relative abundance of the *Lachnospiraceae_NK4A136_group*, *Dubosiella* and *uncultured-Bacteroidales-bacterium* genera in the colon but downregulated the abundance of *Helicobacter* and *Parabacteroides* genera. *Lachnospiraceae_NK4A136_group* is related to butyric acid production (Schirmer et al., 2018), and butyrate has an important protective effect on intestinal homeostasis, which protects the intestinal mucosa and alleviates inflammation (Silva et al., 2018). *Dubosiella* is a producer of short-chain fatty acids (SCFAs) (Ai et al., 2021).The SCFAs, as gut bacterial metabolites, promoted the expression of MUC2 and restored the protective effect of the mucus layer (Li et al., 2021). Several studies detected a decrease in *Dubosiella* in DSS-induced colitis mice (Li et al., 2022). Consistent with this, we found DSS-induced a decrease in the abundance of *Dubosiella*. However, QHCY treatment reversed its decrease. Further spearman correlation analysis showed the abundance of *Dubosiella* was negatively related with the expression of pro-inflammatory cytokines, histological score, and positively correlated with colon length, and the expression of ZO-1 and MUC2. It has been reported that *Parabacteroides* aggravate intestinal inflammation in acute colitis (Dziarski et al., 2016). We also found DSS-induced an increase in the abundance of *Parabacteroides*. However, QHCY treatment reversed its increase. Further spearman correlation analysis showed that the abundance of *Parabacteroides* was positively correlated with pro-inflammatory cytokines, DAI, histological score, and was negatively related with colon length, ZO-1, occludin and MUC2. Overall, these above findings indicated that QHCY could restore gut microbial dysbiosis and regulate intestinal flora in mice with chronic colitis by improving microbial community diversity and regulating microbial community structure and composition. However, the precise underlying potential mechanism of QHCY treatment for chronic colitis should be further investigated.

## 5 Conclusion

QHCY can significantly alleviate clinical symptoms of DSS-induced chronic colitis in mice, protect the function of intestinal mucosal barrier, and improve intestinal microflora by i) inhibiting the expression of TNF-α, IL-6, and IL-1β inflammatory mediators, ii) maintaining the expression of tight junction protein occludin, ZO-1, and MUC2, and iii) improving the diversity of intestinal microflora and regulating the structure and composition of intestinal microflora in mice with chronic colitis. Our findings provide further experimental evidence for the clinical treatment of chronic colitis with QHCY.

## Data Availability

The original contributions presented in the study are included in the article/Supplementary Material, further inquiries can be directed to the corresponding authors.

## Abbreviations

UC, Ulcerative colitis; ZO-1, Zonula occluden-1; MUC2, Mucin-2; TCM, Traditional Chinese medicine; QHCY, Qing Hua Chang Yin; DSS, Dextran sulfate sodium; TNF-α, Tumor necrosis factor-α; IL-1β, Interleukin-1beta; IL-6, Interleukin-6; DAI, Disease activity indices; H&E, Hematoxylin and eosin; PAS, Periodic acid Schiff; IHC, Immunohistochemistry; DAB, Diaminobenzidine; ANOVA, Analysis of variance; OTUs, Operational taxonomic units.

## References

[B1] Actis G. C. Pellicano R. Rosina F. (2015). Inflammatory bowel disease: Traditional knowledge holds the seeds for the future. World J. Gastrointest. Pharmacol. Ther. 6, 10–16. 10.4292/wjgpt.v6.i2.10 25949845 PMC4419088

[B2] Ai X. Wu C. Yin T. Zhur O. Liu C. Yan X. (2021). Antidiabetic function of Lactobacillus fermentum mf423-fermented rice bran and its effect on gut microbiota structure in type 2 diabetic mice. Front. Microbiol. 12, 682290. 10.3389/fmicb.2021.682290 34248898 PMC8266379

[B3] Amasheh M. Grotjohann I. Amasheh S. Fromm A. Söderholm J. D. Zeitz M. (2009). Regulation of mucosal structure and barrier function in rat colon exposed to tumor necrosis factor alpha and interferon gamma *in vitro*: A novel model for studying the pathomechanisms of inflammatory bowel disease cytokines. Scand. J. Gastroenterol. 44, 1226–1235. 10.1080/00365520903131973 19658020

[B4] Cao Q. Lin Y. Yue C. Wang Y. Quan F. Cui X. (2021). IL-6 deficiency promotes colitis by recruiting Ly6C(hi) monocytes into inflamed colon tissues in a CCL2-CCR2-dependent manner. Eur. J. Pharmacol. 904, 174165. 10.1016/j.ejphar.2021.174165 33979652

[B5] Chelakkot C. Ghim J. Ryu S. H. (2018). Mechanisms regulating intestinal barrier integrity and its pathological implications. Exp. Mol. Med. 50, 1–9. 10.1038/s12276-018-0126-x PMC6095905 30115904

[B6] Cui D. J. Yang X. L. Okuda S. Ling Y. W. Zhang Z. X. Liu Q. (2020). Gallincin ameliorates colitis-associated inflammation and barrier function in mice based on network pharmacology prediction. J. Int. Med. Res. 48, 300060520951023. 10.1177/0300060520951023 33322986 PMC7745594

[B7] Cui L. Guan X. Ding W. Luo Y. Wang W. Bu W. (2021). Scutellaria baicalensis Georgi polysaccharide ameliorates DSS-induced ulcerative colitis by improving intestinal barrier function and modulating gut microbiota. Int. J. Biol. Macromol. 166, 1035–1045. 10.1016/j.ijbiomac.2020.10.259 33157130

[B8] Dalal S. R. Chang E. B. (2014). The microbial basis of inflammatory bowel diseases. J. Clin. Investigation 124, 4190–4196. 10.1172/jci72330 PMC4191005 25083986

[B9] Dziarski R. Park S. Y. Kashyap D. R. Dowd S. E. Gupta D. (2016). Pglyrp-regulated gut microflora prevotella falsenii, Parabacteroides distasonis and Bacteroides eggerthii enhance and alistipes finegoldii attenuates colitis in mice. PloS One 11, e0146162. 10.1371/journal.pone.0146162 26727498 PMC4699708

[B10] Fang W. Zhao P. Shen A. Liu L. Chen H. Chen Y. (2021). Effects of Qing Hua Chang Yin on lipopolysaccharide-induced intestinal epithelial tight junction injury in Caco-2 cells. Mol. Med. Rep. 23, 205. 10.3892/mmr.2021.11844 33495820 PMC7821280

[B11] Feuerstein J. D. Moss A. C. Farraye F. A. (2019). Ulcerative colitis. Mayo Clin. Proc. 94, 1357–1373. 10.1016/j.mayocp.2019.01.018 31272578

[B12] Goldsmith J. R. Sartor R. B. (2014). The role of diet on intestinal microbiota metabolism: Downstream impacts on host immune function and health, and therapeutic implications. J. Gastroenterology 49, 785–798. 10.1007/s00535-014-0953-z PMC4035358 24652102

[B13] Gupta R. A. Motiwala M. N. Mahajan U. N. Sabre S. G. (2018). Protective effect of Sesbania grandiflora on acetic acid induced ulcerative colitis in mice by inhibition of TNF-α and IL-6. J. Ethnopharmacol. 219, 222–232. 10.1016/j.jep.2018.02.043 29530609

[B14] Kaminsky L. W. Al-Sadi R. Ma T. Y. (2021). IL-1β and the intestinal epithelial tight junction barrier. Front. Immunol. 12, 767456. 10.3389/fimmu.2021.767456 34759934 PMC8574155

[B15] Ke F. Yadav P. K. Ju L. Z. (2012). Herbal medicine in the treatment of ulcerative colitis. Saudi J. Gastroenterol. 18, 3–10. 10.4103/1319-3767.91726 22249085 PMC3271691

[B16] Ke X. Chen J. Zhang X. Fang W. Yang C. Peng J. (2013a). Qing Hua Chang Yin attenuates lipopolysaccharide-induced inflammatory response in human intestinal cells by inhibiting NF-κB activation. Exp. Ther. Med. 6, 189–193. 10.3892/etm.2013.1071 23935744 PMC3735875

[B17] Ke X. Hu G. Fang W. Chen J. Zhang X. Yang C. (2015). Qing Hua Chang Yin inhibits the LPS-induced activation of the IL-6/STAT3 signaling pathway in human intestinal Caco-2 cells. Int. J. Mol. Med. 35, 1133–1137. 10.3892/ijmm.2015.2083 25633437

[B18] Ke X. Liu L. Zhao P. Youqin C. Peng J. Fang W. (2019). The effects of Qing Hua Chang Yin on the epithelial tight junctions of mice with inflammatory bowel disease. Int. J. Clin. Exp. Med. 12:6864–6873.

[B19] Ke X. Zhou F. Gao Y. Xie B. Hu G. Fang W. (2013b). Qing Hua Chang Yin exerts therapeutic effects against ulcerative colitis through the inhibition of the TLR4/NF-κB pathway. Int. J. Mol. Med. 32, 926–930. 10.3892/ijmm.2013.1458 23900586

[B20] Kelley B. R. Lu J. Haley K. P. Gaddy J. A. Johnson J. G. (2021). Metal homeostasis in pathogenic epsilonproteobacteria: Mechanisms of acquisition, efflux, and regulation. Metallomics 13, mfaa002. 10.1093/mtomcs/mfaa002 33570133 PMC8043183

[B21] Kim N. H. Lee S. M. Kim Y. N. Jeon Y. J. Heo J. D. Jeong E. J. (2020). Standardized fraction of turbinaria ornata alleviates dextran sulfate sodium-induced chronic colitis in C57bl/6 mice via upregulation of FOXP3(+) regulatory T cells. Biomolecules 10, 1463. 10.3390/biom10101463 33092149 PMC7590162

[B22] Li D. P. Cui M. Tan F. Liu X. Y. Yao P. (2021). High red meat intake exacerbates dextran sulfate-induced colitis by altering gut microbiota in mice. Front. Nutr. 8, 646819. 10.3389/fnut.2021.646819 34355008 PMC8329097

[B23] Li Q. Chen G. Zhu D. Zhang W. Qi S. Xue X. (2022). Effects of dietary phosphatidylcholine and sphingomyelin on DSS-induced colitis by regulating metabolism and gut microbiota in mice. J. Nutr. Biochem. 105, 109004. 10.1016/j.jnutbio.2022.109004 35351615

[B24] Lin J. C. Wu J. Q. Wang F. Tang F. Y. Sun J. Xu B. (2019). QingBai decoction regulates intestinal permeability of dextran sulphate sodium-induced colitis through the modulation of notch and NF-κB signalling. Cell Prolif. 52, e12547. 10.1111/cpr.12547 30657238 PMC6496276

[B25] Melhem H. Regan-Komito D. Niess J. H. (2021). Mucins dynamics in physiological and pathological conditions. Int. J. Mol. Sci. 22, 13642. 10.3390/ijms222413642 34948435 PMC8707880

[B26] Ng S. C. Shi H. Y. Hamidi N. Underwood F. E. Tang W. Benchimol E. I. (2017). Worldwide incidence and prevalence of inflammatory bowel disease in the 21st century: A systematic review of population-based studies. Lancet 390, 2769–2778. 10.1016/s0140-6736(17)32448-0 29050646

[B27] Nishida A. Inoue R. Inatomi O. Bamba S. Naito Y. Andoh A. (2018). Gut microbiota in the pathogenesis of inflammatory bowel disease. Clin. J. Gastroenterol. 11, 1–10. 10.1007/s12328-017-0813-5 29285689

[B28] Quévrain E. Maubert M. A. Michon C. Chain F. Marquant R. Tailhades J. (2016). Identification of an anti-inflammatory protein from Faecalibacterium prausnitzii, a commensal bacterium deficient in Crohn's disease. Gut 65, 415–425. 10.1136/gutjnl-2014-307649 26045134 PMC5136800

[B29] Ryan F. J. Ahern A. M. Fitzgerald R. S. Laserna-Mendieta E. J. Power E. M. Clooney A. G. (2020). Colonic microbiota is associated with inflammation and host epigenomic alterations in inflammatory bowel disease. Nat. Commun. 11, 1512. 10.1038/s41467-020-15342-5 32251296 PMC7089947

[B30] Schirmer M. Franzosa E. A. Lloyd-Price J. McIver L. J. Schwager R. Poon T. W. (2018). Dynamics of metatranscription in the inflammatory bowel disease gut microbiome. Nat. Microbiol. 3, 337–346. 10.1038/s41564-017-0089-z 29311644 PMC6131705

[B31] Shen P. Zhang Z. He Y. Gu C. Zhu K. Li S. (2018). Magnolol treatment attenuates dextran sulphate sodium-induced murine experimental colitis by regulating inflammation and mucosal damage. Life Sci. 196, 69–76. 10.1016/j.lfs.2018.01.016 29355546

[B32] Silva J. P. B. Navegantes-Lima K. C. Oliveira A. L. B. Rodrigues D. V. S. Gaspar S. L. F. Monteiro V. V. S. (2018). Protective mechanisms of butyrate on inflammatory bowel disease. Curr. Pharm. Des. 24, 4154–4166. 10.2174/1381612824666181001153605 30277149

[B33] Wang X. Fan F. Cao Q. (2016). Modified Pulsatilla decoction attenuates oxazolone-induced colitis in mice through suppression of inflammation and epithelial barrier disruption. Mol. Med. Rep. 14, 1173–1179. 10.3892/mmr.2016.5358 27278299 PMC4940073

[B34] Wirtz S. Popp V. Kindermann M. Gerlach K. Weigmann B. Fichtner-Feigl S. (2017). Chemically induced mouse models of acute and chronic intestinal inflammation. Nat. Protoc. 12, 1295–1309. 10.1038/nprot.2017.044 28569761

[B35] Wu F. Shao Q. Hu M. Zhao Y. Dong R. Fang K. (2020). Wu-Mei-Wan ameliorates chronic colitis-associated intestinal fibrosis through inhibiting fibroblast activation. J. Ethnopharmacol. 252, 112580. 10.1016/j.jep.2020.112580 31972322

[B36] Wu Y. Tang L. Wang B. Sun Q. Zhao P. Li W. (2019). The role of autophagy in maintaining intestinal mucosal barrier. J. Cell Physiol. 234, 19406–19419. 10.1002/jcp.28722 31020664

[B37] Xu Z. Chen W. Deng Q. Huang Q. Wang X. Yang C. (2020). Flaxseed oligosaccharides alleviate DSS-induced colitis through modulation of gut microbiota and repair of the intestinal barrier in mice. Food and Funct. 11, 8077–8088. 10.1039/d0fo01105c 32856645

[B38] Yadav V. Varum F. Bravo R. Furrer E. Bojic D. Basit A. W. (2016). Inflammatory bowel disease: Exploring gut pathophysiology for novel therapeutic targets. Transl. Res. J. Laboratory Clin. Med. 176, 38–68. 10.1016/j.trsl.2016.04.009 27220087

[B39] Yamada S. Kanda Y. (2021). Evaluation of barrier functions in human iPSC-derived intestinal epithelium. Methods Mol. Biol. 2367, 27–35. 10.1007/7651_2021_346 33661485

[B40] Yao D. Dai W. Dong M. Dai C. Wu S. (2021a). MUC2 and related bacterial factors: Therapeutic targets for ulcerative colitis. EBioMedicine 74, 103751. 10.1016/j.ebiom.2021.103751 34902790 PMC8671112

[B41] Yao H. Shi Y. Yuan J. Sa R. Chen W. Wan X. (2021b). Matrine protects against DSS-induced murine colitis by improving gut barrier integrity, inhibiting the PPAR-α signaling pathway, and modulating gut microbiota. Int. Immunopharmacol. 100, 108091. 10.1016/j.intimp.2021.108091 34474274

