# Supplementary material for: Qing Hua Chang Yin alleviates chronic colitis of mice by protecting intestinal barrier function and improving colonic microflora
Source: Front Pharmacol. 2023 Jul 27;14:1176579. doi: 10.3389/fphar.2023.1176579 (PMC10413571; doi:10.3389/fphar.2023.1176579)
Supplement: Supplementary file 3 [file DataSheet1.PDF]

Fig.3A

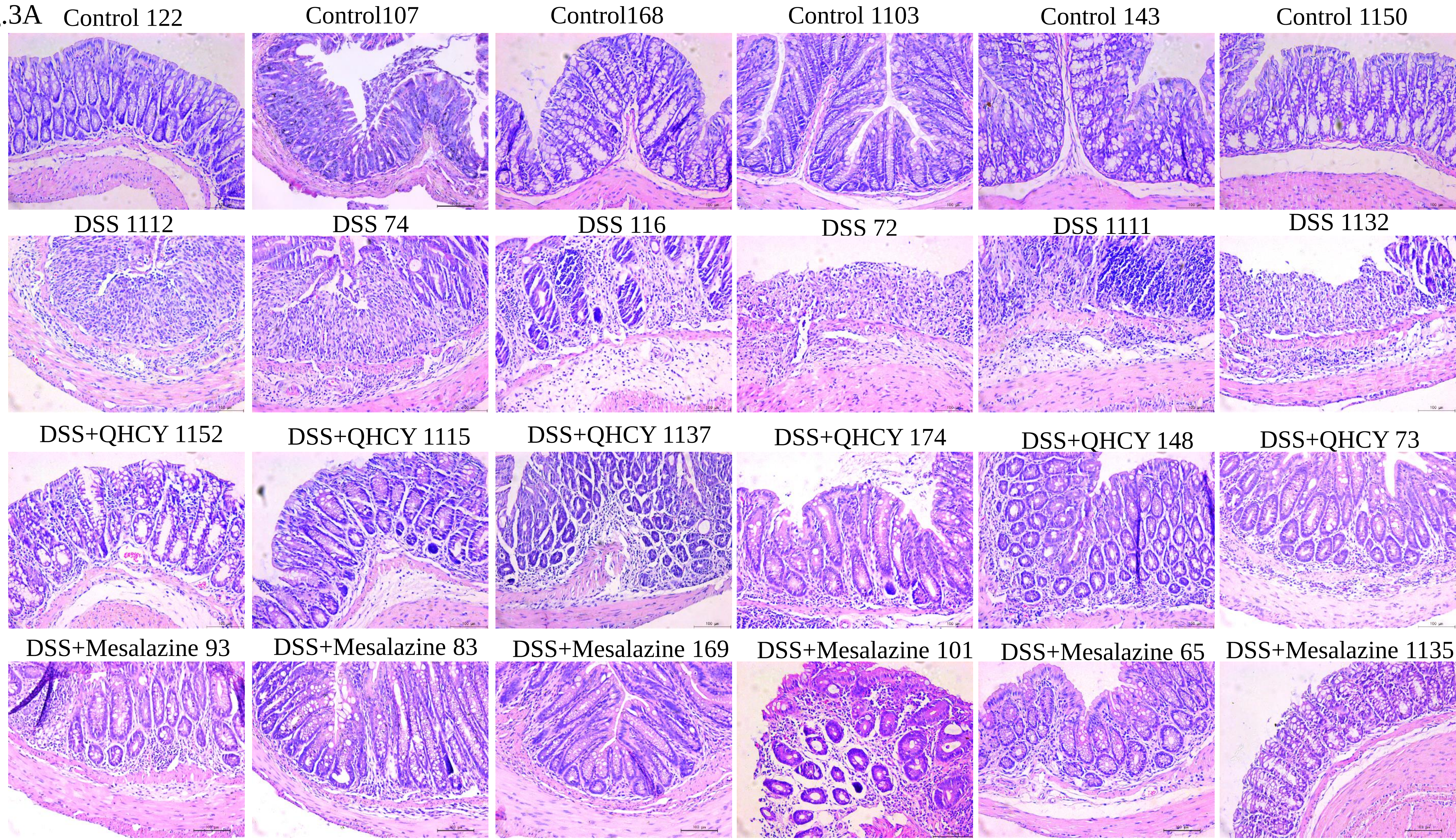
Control 122
Control107
Control168
Control 1103
Control 143
Control 1150
DSS 1112
DSS 74
DSS 116
DSS 72
DSS 1111
DSS 1132
DSS+QHCY 1152
DSS+QHCY 1115
DSS+QHCY 1137
DSS+QHCY 174
DSS+QHCY 148
DSS+QHCY 73
DSS+Mesalazine 93
DSS+Mesalazine 83
DSS+Mesalazine 169
DSS+Mesalazine 101
DSS+Mesalazine 65
DSS+Mesalazine 1135

Fig.3C

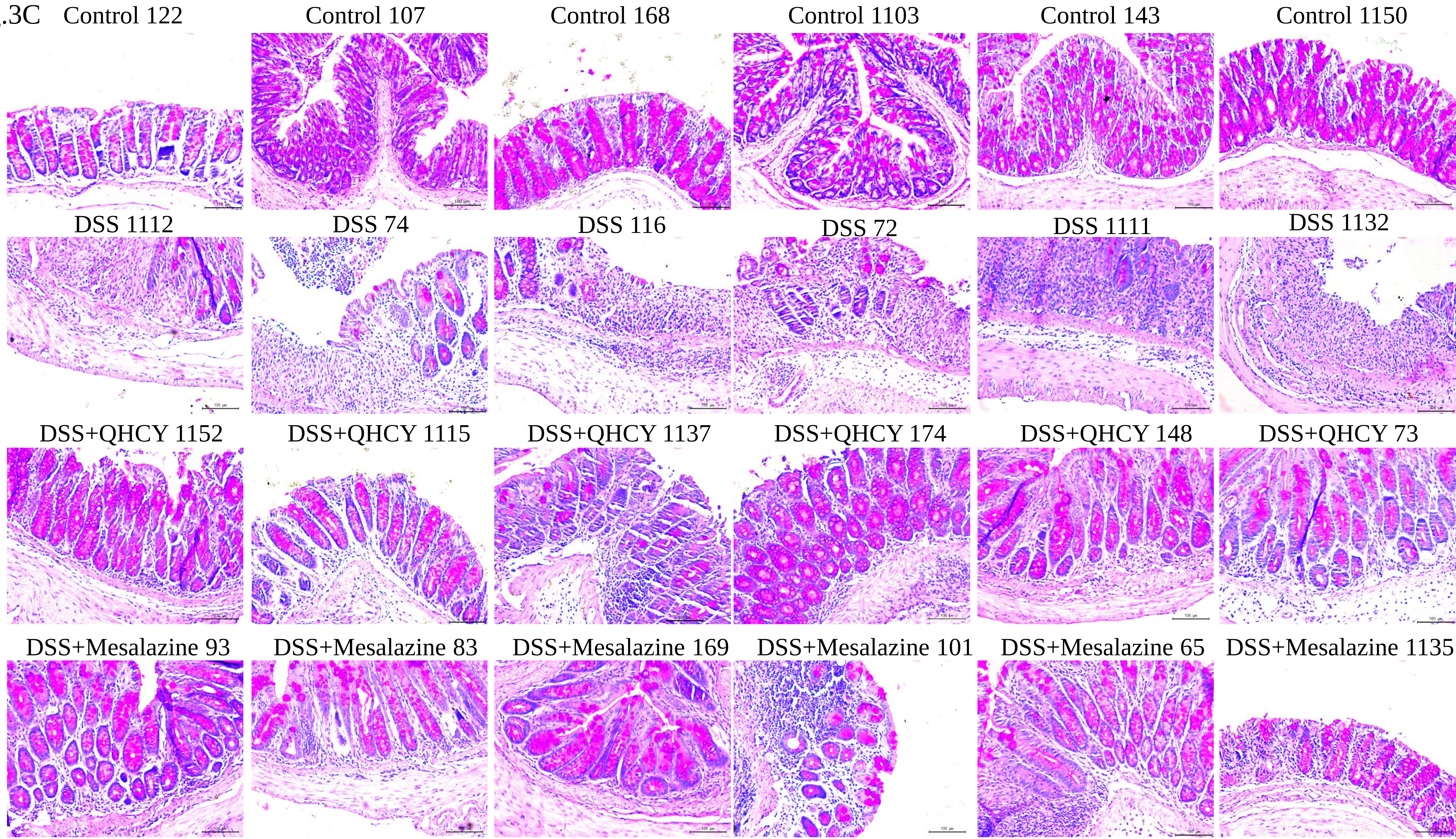
Control 122
Control 107
Control 168
Control 1103
Control 143
Control 1150
DSS 1112
DSS 74
DSS 116
DSS 72
DSS 1111
DSS 1132
DSS+QHCY 1152
DSS+QHCY 1115
DSS+QHCY 1137
DSS+QHCY 174
DSS+QHCY 148
DSS+QHCY 73
DSS+Mesalazine 93
DSS+Mesalazine 83
DSS+Mesalazine 169
DSS+Mesalazine 101
DSS+Mesalazine 65
DSS+Mesalazine 1135

Fig.4A

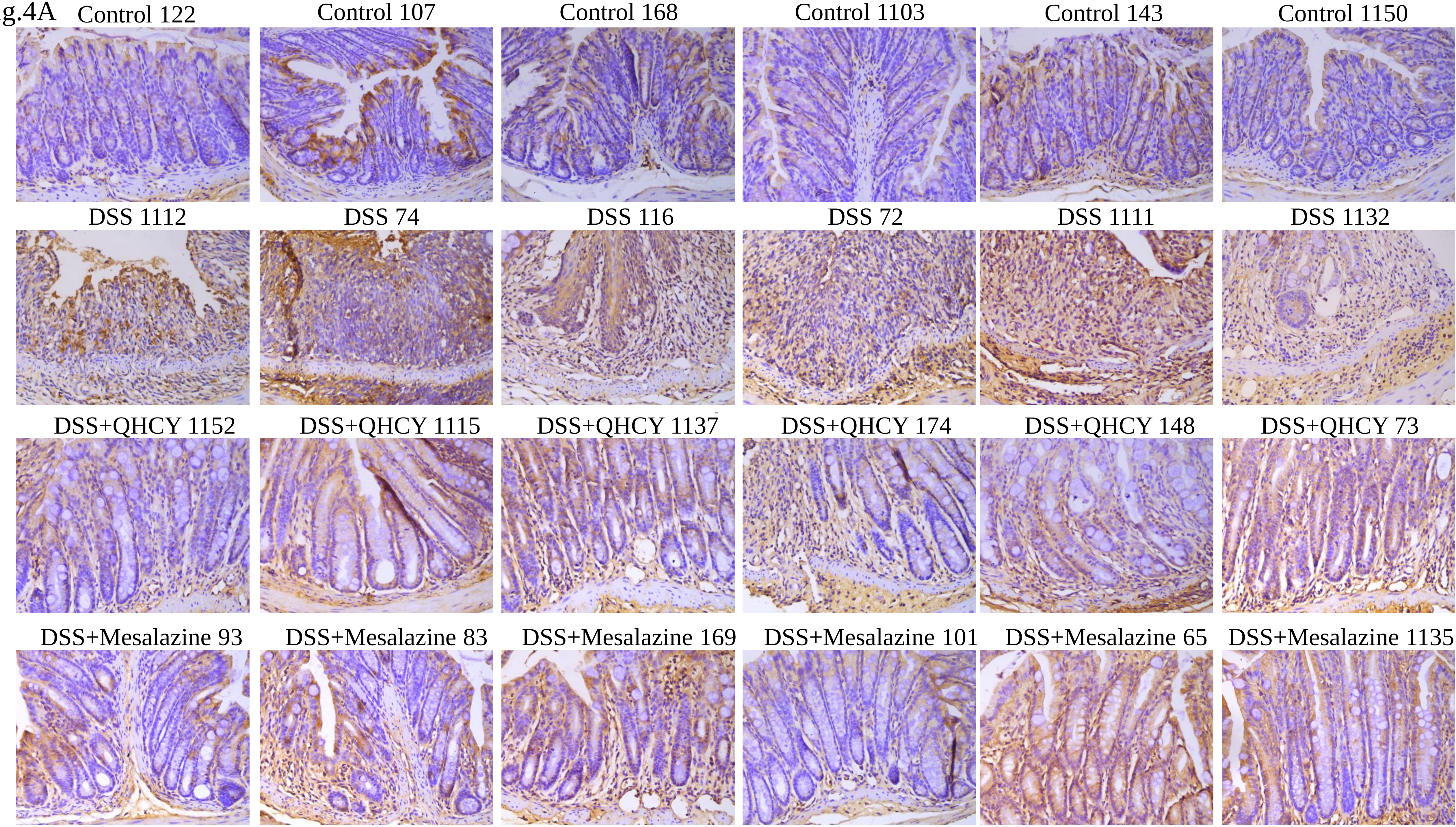
Control 122
Control 107
Control 168
Control 1103
Control 143
Control 1150
DSS 1112
DSS 74
DSS 116
DSS 72
DSS 1111
DSS 1132
DSS+QHCY 1152
DSS+QHCY 1115
DSS+QHCY 1137
DSS+QHCY 174
DSS+QHCY 148
DSS+QHCY 73
DSS+Mesalazine 93
DSS+Mesalazine 83
DSS+Mesalazine 169
DSS+Mesalazine 101
DSS+Mesalazine 65
DSS+Mesalazine 1135

Fig.4B

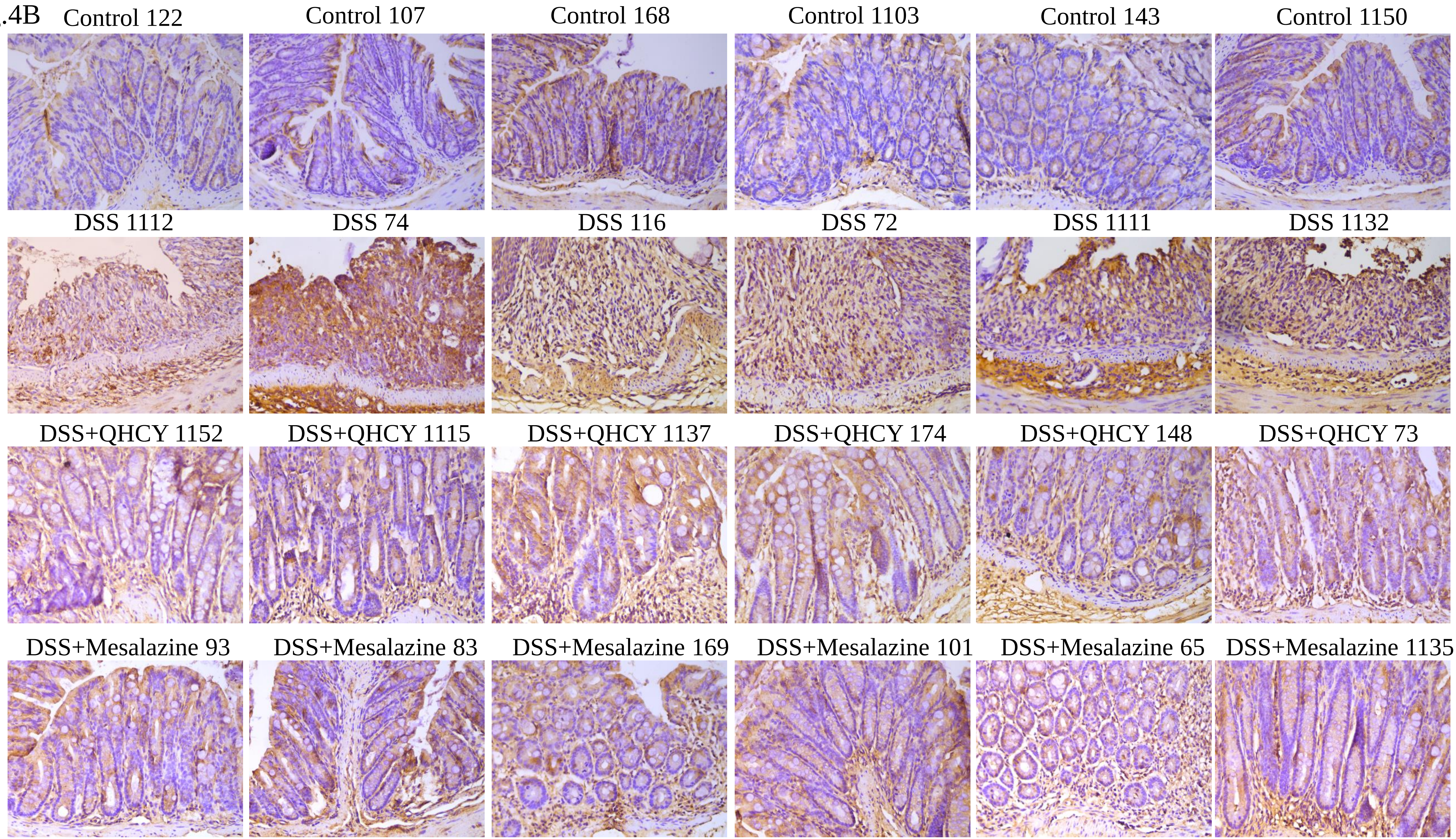
Control 122
Control 107
Control 168
Control 1103
Control 143
Control 1150
DSS 1112
DSS 74
DSS 116
DSS 72
DSS 1111
DSS 1132
DSS+QHCY 1152
DSS+QHCY 1115
DSS+QHCY 1137
DSS+QHCY 174
DSS+QHCY 148
DSS+QHCY 73
DSS+Mesalazine 93
DSS+Mesalazine 83
DSS+Mesalazine 169
DSS+Mesalazine 101
DSS+Mesalazine 65
DSS+Mesalazine 1135

Fig.4C

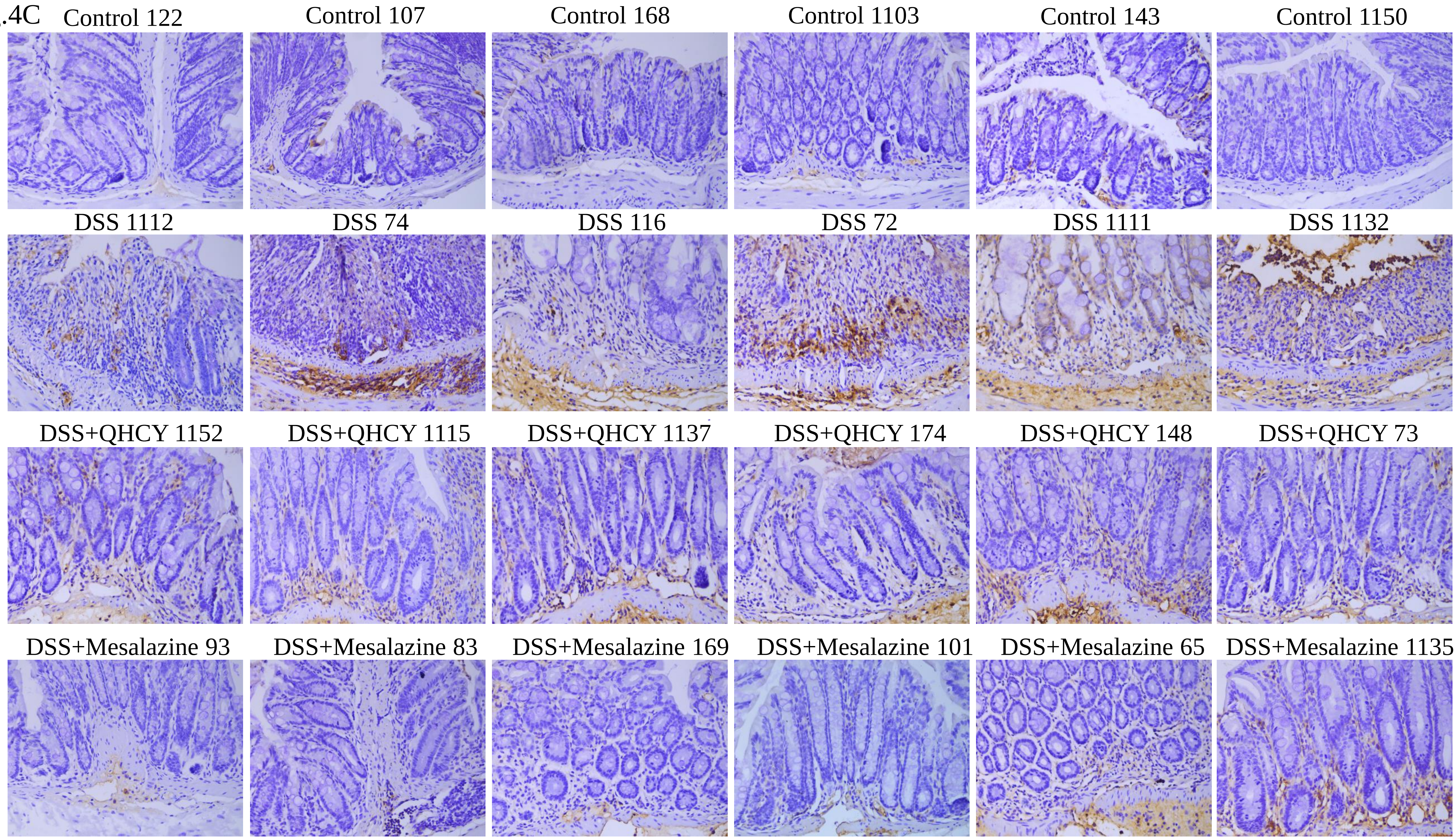
Control 122
Control 107
Control 168
Control 1103
Control 143
Control 1150
DSS 1112
DSS 74
DSS 116
DSS 72
DSS 1111
DSS 1132
DSS+QHCY 1152
DSS+QHCY 1115
DSS+QHCY 1137
DSS+QHCY 174
DSS+QHCY 148
DSS+QHCY 73
DSS+Mesalazine 93
DSS+Mesalazine 83
DSS+Mesalazine 169
DSS+Mesalazine 101
DSS+Mesalazine 65
DSS+Mesalazine 1135

Fig.5A

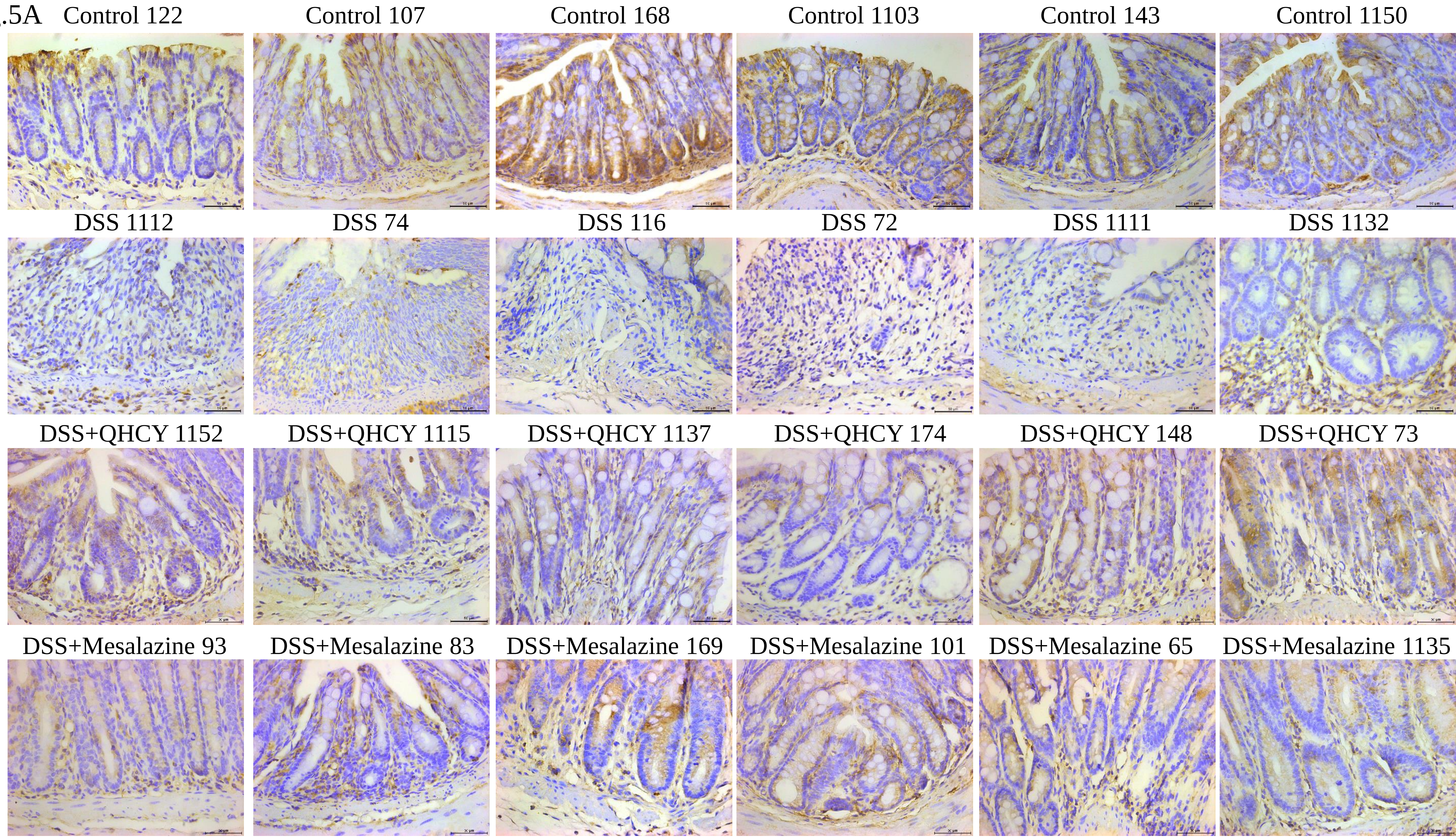
Control 122
Control 107
Control 168
Control 1103
Control 143
Control 1150
DSS 1112
DSS 74
DSS 116
DSS 72
DSS 1111
DSS 1132
DSS+QHCY 1152
DSS+QHCY 1115
DSS+QHCY 1137
DSS+QHCY 174
DSS+QHCY 148
DSS+QHCY 73
DSS+Mesalazine 93
DSS+Mesalazine 83
DSS+Mesalazine 169
DSS+Mesalazine 101
DSS+Mesalazine 65
DSS+Mesalazine 1135

Fig.5B

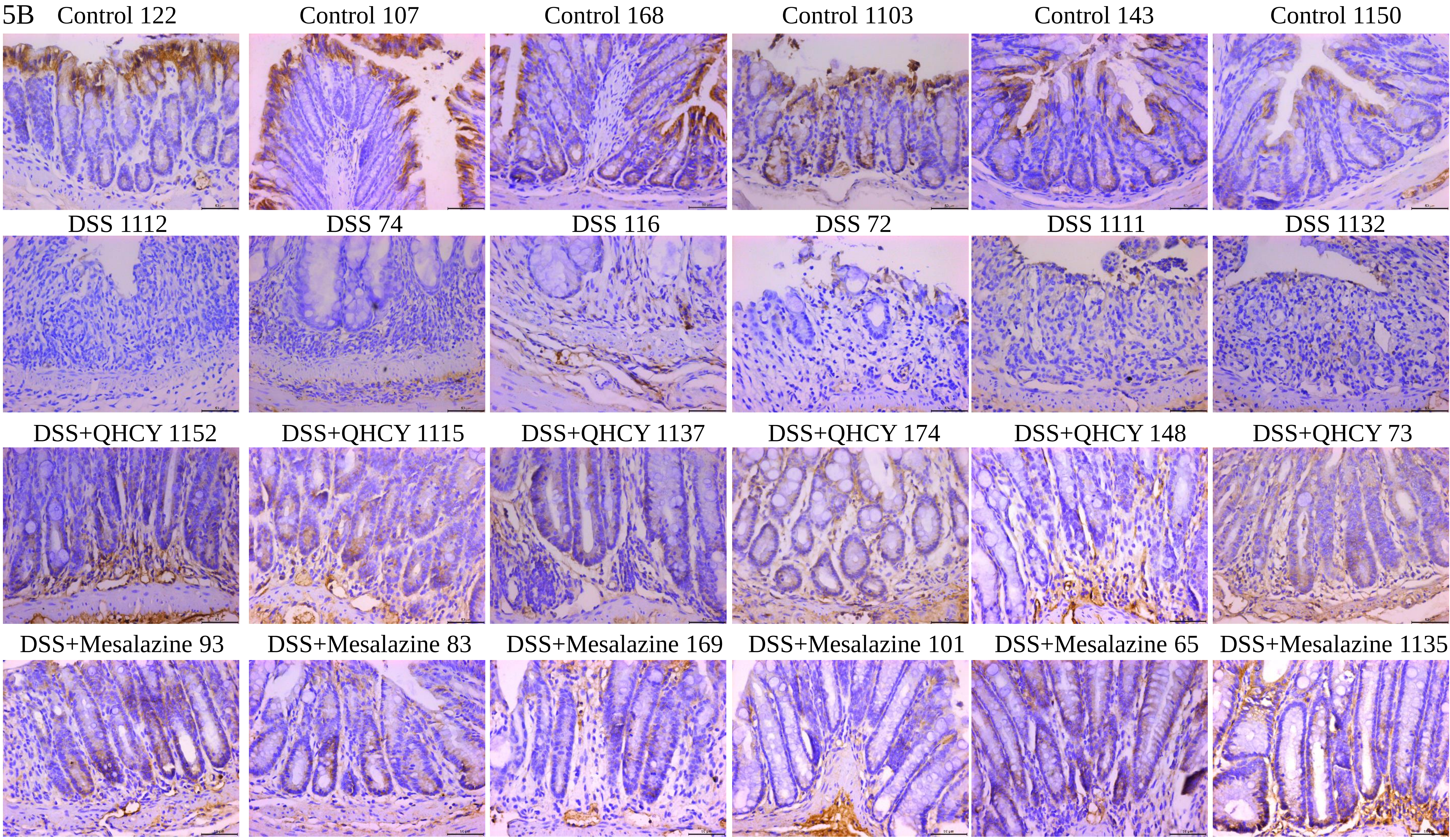
Control 122
Control 107
Control 168
Control 1103
Control 143
Control 1150
DSS 1112
DSS 74
DSS 116
DSS 72
DSS 1111
DSS 1132
DSS+QHCY 1152
DSS+QHCY 1115
DSS+QHCY 1137
DSS+QHCY 174
DSS+QHCY 148
DSS+QHCY 73
DSS+Mesalazine 93
DSS+Mesalazine 83
DSS+Mesalazine 169
DSS+Mesalazine 101
DSS+Mesalazine 65
DSS+Mesalazine 1135

Fig.5C

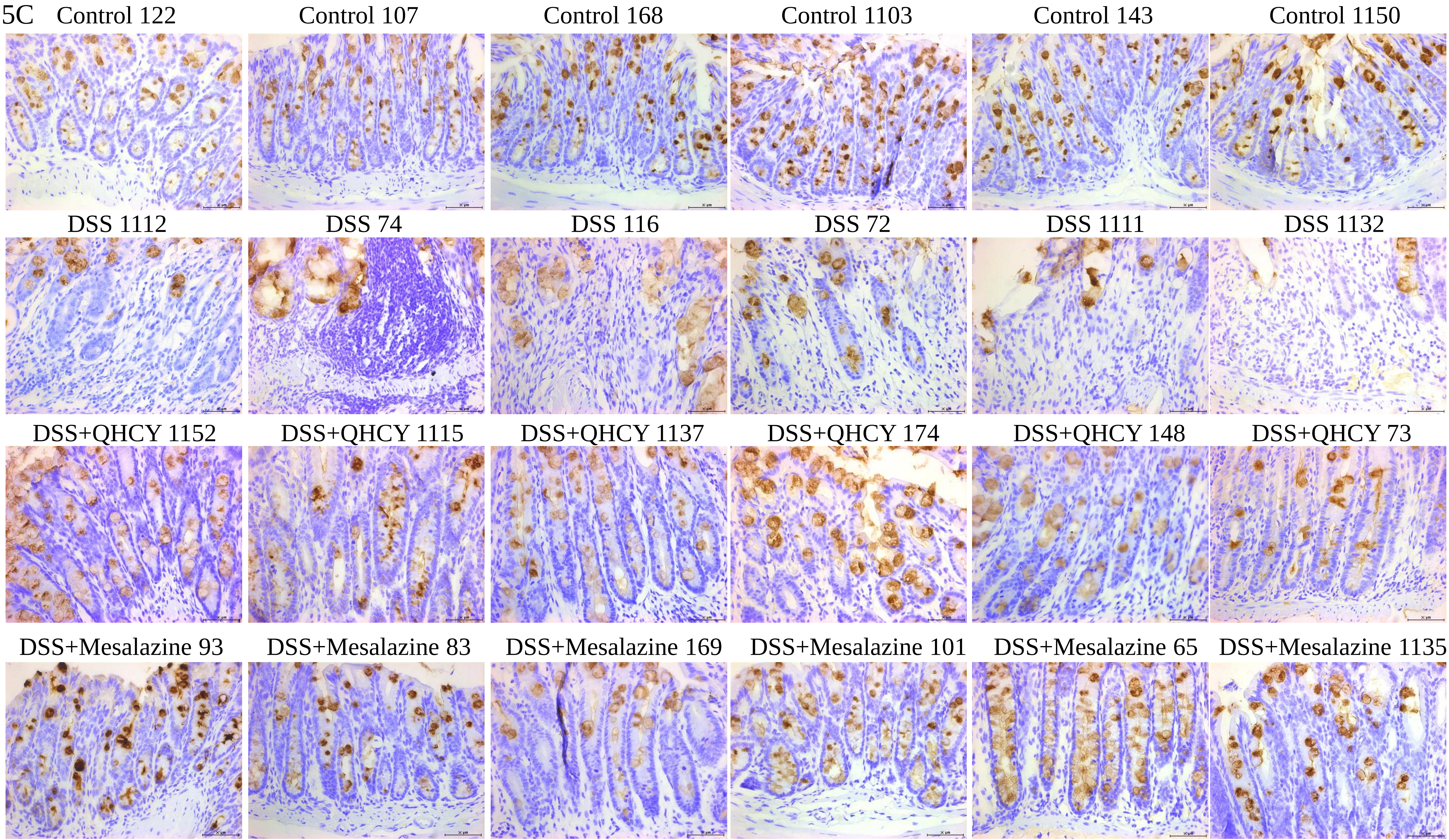
Control 122
Control 107
Control 168
Control 1103
Control 143
Control 1150
DSS 1112
DSS 74
DSS 116
DSS 72
DSS 1111
DSS 1132
DSS+QHCY 1152
DSS+QHCY 1115
DSS+QHCY 1137
DSS+QHCY 174
DSS+QHCY 148
DSS+QHCY 73
DSS+Mesalazine 93
DSS+Mesalazine 83
DSS+Mesalazine 169
DSS+Mesalazine 101
DSS+Mesalazine 65
DSS+Mesalazine 1135
