# Supplementary material for: Qing Hua Chang Yin alleviates chronic colitis of mice by protecting intestinal barrier function and improving colonic microflora
Source: Front Pharmacol. 2023 Jul 27;14:1176579. doi: 10.3389/fphar.2023.1176579 (PMC10413571; doi:10.3389/fphar.2023.1176579)
Supplement: Supplementary file 4 [file Table5.DOCX]

Supplementary Material

# Supplementary Table

**Table1. Disease activity index(DAI)**

| **Index** | **Weight loss(%)** | **Stool** | **Crypt damage** |
| --- | --- | --- | --- |
| 0 | None | Well-formed pellets | None |
| 1 | 1-5 | - | - |
| 2 | 6-10 | Pasty and semiformed | Positive bleeding |
| 3 | 11-20 | - | - |
| 4 | >20 | Liquid | Gross bleeding |

**Table2.** **Histological grading of colitis in DSS-induced colitis mice**

| **Score** | **Inflammation** | **Extent** | **Crypt damage** | **Percent involvement(%)** |
| --- | --- | --- | --- | --- |
| 0 | None | None | None | None |
| 1 | Slight | Mucosa | Basal1/3damaged | 1-25% |
| 2 | Moderate | Mucosa and sub-mucosa | Basal2/3damaged | 26-50% |
| 3 | Severe | Transmural | Only surface epithelium intact | 51-75% |
| 4 |  |  | Entire crypt and epithelium lost | 76-100% |
